# Acromegaly disease activity according to ACRODAT®, a cross-sectional study in Spain: ACROVAL study

**DOI:** 10.1007/s12020-021-02900-0

**Published:** 2021-10-19

**Authors:** Mónica Marazuela, Concepción Blanco, Ignacio Bernabeu, Edelmiro Menendez, Rocío Villar, Miguel Paja, Miguel Sampedro-Nuñez, M. Luz Samaniego, Marcos Díaz-Muñoz, Laura Sánchez-Cenizo

**Affiliations:** 1 Hospital Universitario de La Princesa, Madrid, Universidad Autónoma de Madrid, Madrid, Spain; 2 Hospital Universitario Príncipe de Asturias, Alcalá de Henares, Madrid Spain; 3 Complejo Hospitalario Universitario de Santiago de Compostela, Santiago de Compostela, La Coruña Spain; 4 Hospital Universitario Central de Asturias (HUCA), Instituto de investigación del Principado de Asturias (ISPA), Oviedo, Asturias Spain; 5 Hospital Universitario de Basurto, Bilbao, Vizcaya Spain; 6 TFS Statistical Services, Madrid, Madrid Spain; 7 Pfizer S.L.U., Alcobendas, Madrid Spain

**Keywords:** ACRODAT®, Acromegaly, IGF-I, PASQ, Disease Activity, Quality of life, AcroQoL

## Abstract

**Objectives:**

To evaluate disease activity status using the Acromegaly Disease Activity Tool (ACRODAT^®^) in a cohort of Spanish acromegaly patients, to assess the relationship between the level of disease activity according to both ACRODAT^®^ and the physicians’ clinical evaluation, and to study the potential discrepancies in the perception of symptoms between physicians and patients.

**Design:**

Multicenter, observational, descriptive and cross-sectional study.

**Methods:**

Disease activity was assessed in adult patients with acromegaly under pharmacological treatment during at least 6 months using ACRODAT^®^.

**Results:**

According to ACRODAT^®^, 48.2%, 31.8% and 20.0% of a total of 111 patients were classified as having a stable disease (S), mild disease activity (M-DA) and significant disease activity (S-DA) respectively. ACRODAT^®^ classification of disease activity significantly correlated with physicians’ opinion, with a moderate inter-rater agreement and a specificity of 92.45% (PPV = 86.21%). No correlation was found between IGF-I levels and severity of symptoms or quality of life (QoL). A decision to take clinical action was significantly more frequent in S-DA and M-DA patients than S patients but no action was taken on 5 (22.7%) and 27 (77.1%) S-DA and M-DA patients, respectively

**Conclusions:**

ACRODAT^®^ detected disease activity in 51.8% of patients. Interestingly, although M-DA and S-DA patients were likely to be in the process of being controlled, action was not always taken on these patients. ACRODAT^®^ is a validated and highly specific tool that may be useful to routinely monitor acromegaly and to identify patients with non-obvious disease activity by incorporating “patient-centred” parameters like symptoms and QoL to the clinical evaluation of acromegaly.

## Introduction

Acromegaly, a rare and chronic disease usually caused by a pituitary adenoma, is characterized by hyper-secretion of growth hormone (GH) with a consequent increase in insulin-like growth factor I (IGF-I) [1, 2].

Acromegaly is associated with multiple co-morbidities, premature mortality, and physical disfigurement [1]. The most serious health consequences include type 2 diabetes, high blood pressure, increased risk of cardiovascular diseases, arthropathy and sleep apnoea [3, 4]. In fact, the presence at the diagnosis of diabetes mellitus, cardiovascular disease and hypertension are significantly associated with reduced survival [5]. In addition, acromegaly patients experience decreased energy and psychological disturbances (loss of initiative, mood lability, impaired self-esteem, depression and anxiety) that significantly affect their quality of life (QoL) [6–9].

The goals of acromegaly treatment are to achieve overall long-term biochemical control, control tumour mass and decrease the risk of developing systemic comorbidities, thereby reducing mortality [10, 11].. Even when biochemical control is achieved, many patients still suffer from physical and psychological residual morbidity that can lead to persistently impaired QoL [6, 12–14]. In fact, the correlation between “biochemical” severity (measured by IGF-I levels) and impact of disease on patient’s lives is weak [14, 15]. In addition, from the patient’s perspective, symptoms and QoL are critical parameters of disease control [6, 12], therefore they should be assessed in clinical practice for an adequate long-term management of acromegaly [13].

Discrepancy between patient’s and physician’s ratings of general health status has been previously demonstrated [6, 16, 17]. The consequence of such a discordant viewpoint regarding disease activity is that decisions are often prone to not being shared between patients and physicians [16]. Acromegaly patients place more importance on patient-centred parameters (i.e., signs/symptoms, comorbid conditions and QoL) than do expert endocrinologists [6]. Therefore, the patients’ own perspectives of their health status may be an important additional measure to assess the level of disease activity and support clinical decision-making and, together with IGF-I level and tumour status could provide a more accurate assessment of the disease status [6, 18]

To aid in the global clinical management of acromegaly, a multidimensional clinical decision support tool, the Acromegaly Disease Activity Tool (ACRODAT^®^) was developed [18]. The ACRODAT^®^ software medical device is a disease specific tool that allows the classification of acromegaly disease activity using a multidimensional approach in three categories: stable (S, adequately controlled), mild disease activity (M-DA: further evaluation of the patient’s condition is needed), or significant disease activity (S-DA: clinical action is required) [18]. The development of this tool was based on the identification of five key health status parameters in acromegaly and the definition of three levels of severity for each of them: IGF-I level, tumour status, presence of co-morbidities (cardiac disease, diabetes, sleep apnoea), signs and symptoms and health-related QoL (Table 1). A scoring algorithm was built based on the classification of 243 hypothetical cases allowing the classification of the disease in the three above mentioned categories [18].

**Table 1:** ACRODAT® disease activity categories

Parameters	Disease activity Level		
	S	M-DA	S-DA
IGF-I levels	Within normal limits	Exceeds the ULN but not >1.2 ULN, or is below LLN	> 1.2 ULN
Tumour status	Tumour is not visible or has not changed since prior MRI	A slight increase in tumour size (≤20%) since prior MRI	A significant increase in tumour size (>20%) since prior MRI and/or tumour invasiveness and/or worsening in vision
Comorbidities*	Well controlled: No diabetes, apnoea and cardiac disease or, if present, well controlled by therapy	Partially controlled: Diabetes is well controlled by therapy, no apnoea, no cardiac disease (or, if present, well controlled by therapy) or no diabetes but presence of apnoea and/or cardiac disease not well controlled by therapy	Non-controlled: Diabetes is not well controlled by therapy and presence of moderate/severe apnoea and /or uncontrolled cardiac disease
Symptoms (PASQ)	Mild: patient reports no or only mild symptoms (all rated ≤ 2)	Moderate: patients report presence of some symptoms, but no single symptoms exceed a score of 6, and the mean score is ≤ 4 overall	Severe: patients report symptoms with a mean score >4 or one or more symptoms rated >6
QoL (AcroQoL)	Score≥ 60: No or minimal impairment in QoL	40≤ score>60: mild to moderate impairment in QoL	Score<40: significant impairment in QoL

*S* stable, *M-DA* mild disease activity, *S-DA* significant disease activity, *IGF-I* insulin-like growth factor-I, *ULN* upper limit of normal, *LLN* lower limit of normal, *MRI* magnetic resonance imaging; Comorbidities*: cardiac disease (including hypertension, hyperlipidaemia or other cardiac abnormalities), diabetes, sleep apnoea; PASQ: Patient acromegalic symptom questionnaire; AcroQoL: the Acromegaly Quality of life Questionnaire

Modified from Van der Lely AJ, et al. Pituitary 2017 Dec; 20 (6): 692–701

Due to the recent development of the tool, its clinical application has not been studied yet. This study was aimed to evaluate disease activity status using ACRODAT^®^ in a cohort of Spanish patients with acromegaly. Other secondary objectives included the assessment of the relationship between patient demographic characteristics and ACRODAT^®^ disease categories; to assess the relation between classification of disease activity by ACRODAT^®^ and by physician criteria and to study the potential discrepancies in the perception of symptoms between physicians and patients.

## Materials and methods

ACROVAL was an observational, cross-sectional, multicenter study conducted in 12 representative hospitals from all over Spain. The study included adult patients diagnosed with acromegaly, on pharmacological treatment for at least 6 months, and with complete clinical reports available. All patients provided their informed written consent. The study was approved by the Clinical Research Ethics Committee of every participant site and it was conducted in accordance with the Declaration of Helsinki principles.

### Data collection

Demographic data, acromegaly clinical history, co-morbidities, IGF-I levels and tumour status were obtained from clinical records. Quality of life (QoL), severity of symptoms, job status, disease activity and decision taken regarding acromegaly treatment (surgery, radiotherapy, change in drug treatment or no action) were collected at the study visit. IGF-I levels, tumour status and therapeutic decision at the visit before study visit was also collected from clinical records.

QoL was determined by the *Acromegaly Quality of life Questionnaire* (AcroQoL) [19]. The AcroQoL questionnaire [19] is a 22-item, disease-specific QoL tool. Each question has five possible answers scored 1–5, with a total maximum score of 110. The score of 110 reflects the best possible QoL. AcroQoL overall score and subscores included in the AcroQoL questionnaire were normalized (0–100) being 100 the best possible QoL.

Severity of symptoms was quantified using the *Patient-Assessed Acromegaly Symptom Questionnaire (PASQ)* [20]. The PASQ [12] comprises six questions that evaluate six acromegaly key symptoms. Each item is scored on a 9-point scale (0 no symptoms-8 severe incapacitating symptoms). The total PASQ score is the sum of the individual symptom scores (maximum = 48). An additional seventh question addresses the overall health status, which was scored ranging from 0 (best possible) to 10 (worst possible). The questionnaire was completed by physicians (phPASQ) and by patients (paPASQ) in order to compare both perspectives. phPASQ and paPASQ were mutually blinded. Symptoms status are measured in ACRODAT^®^ by a Signs and Symptoms Score (SSS), an abbreviated version of the PASQ score that omits the numbness or tingling of the extremities and the overall health status questions [18]. IGF-I level was measured locally and recorded as a proportion coefficient x the upper limit of normal (ULN) for the respective method used.

Tumour status was classified by the physician in the same three categories that use ACRODAT^®^ (Table 1).

Comorbidities were assigned based on the presence or absence and severity of diabetes, sleep apnoea and cardiac disease (hypertension, hyperlipidaemia, or other cardiac abnormalities) [18] (Table 1).

The ACRODAT^®^ tool was used to assess disease activity, entering the data collected in the study. According to the algorithm behind ACRODAT^®^, IGF-I and tumour status are the predominant parameters in the classification of M-DA or S-DA. Only when the IGF-I level is ≤1.2 x ULN and tumour size did not significantly increase, the remaining three parameters contribute to the classification in a compensatory manner. Data were entered into the ACRODAT tool after the patent’s visits.

Disease activity level (stable, moderate disease activity and significant disease activity) according to physician’s point of view was also recorded. The disease activity was assessed by an endocrinologist with specific experience in acromegaly and neuroendocrinology and the disease activity level was classified according to their experience and criteria (endocrinologist had access to IGF-I levels, tumour status, phPASQ and comorbidities).

### Statistical methodology

A descriptive statistical analysis of all the variables was performed, including central tendency and dispersion measures for continuous variables, and absolute and relative frequencies for categorical variables. Student’s *t* test, Mann–Whitney-U test or Kruskall Wallis H test were used to compare quantitative variables and Pearson’s chi-square or Fisher’s exact tests for qualitative variables. The assumptions of normality distribution (Kolmogorov-Smirnov and Shapiro-Wilk) and homoscedasticity test (Levene’s test) of the different variables were studied for the use of parametric tests.

Pearson/Spearman correlation coefficient was used to study the association between the ACRODAT® scale score and the rest of the variables of analysis, as well as between the variables phPASQ vs paPASQ, IGF-I vs phPASQ and IGF-I vs paPASQ. The Kappa agreement coefficient was calculated to test concordance of disease activity status according to ACRODAT^®^ vs. physicians’ clinical evaluation and to test the concordance between paPASQ and phPASQ. Predictive analysis of ACRODAT^®^ was assessed by determining the values of sensitivity, specificity, the positive predictive value (PPV), and negative (NPV) predictive value. The gold standard used as a reference was the physicians’ clinical evaluation. Tests were two-tailed with a significance level of 5%. The p-values obtained between discrepancies in disease activity and the clinical characteristics of the patients were calculated through a univariate logistic regression model whose dependent variable is the agreement between ACRODAT® classifications and the physiscian’s perception (yes/no). The independent variables are summarized in Supplementary Table 1. In order to determine the factors associated with the existence of discrepancies in the degree of acromegaly activity according to the ACRODAT® scale and the perception of physician, a multivariate regression model was carried out, whose dependent variable be the agreement between both criteria (without discrepancies vs with discrepancies) and as possible associated factors all those that are significant in the model univariate (*p* value < 0.250). Data were analyzed using SPSS 19.0.

## Results

### Patient characteristics

In total, 113 patients from 12 centres were included, of whom 111 were considered evaluable in the analysis. Two patients were excluded because their duration of pharmacological treatment was less than 6 months. The acromegaly activity according to the ACRODAT® classification was not available for 1 patient.Table 2 summarizes demographic and clinical characteristics of patients. Macroadenoma was present in most patients (82.7%) at diagnosis (Table 2). However, in 97.1% of patients, tumour was not visible or had not changed in volume since prior MRI and only 3 patients (2.9%) had experienced a slight increase (≤20%) since prior MRI (Table 2). IGF-I levels were within normal limits in 64.0% of patients, above ULN but below 1.2 ULN in 16.2% of patients and above 1.2 ULN in 19.8% of patients.

**Table 2:** Demographic and clinical characteristics

	Total	S	M-DA	S-DA	*p* value
	111	53	35	2	
Women, *n* (%)	57 (51.4)	26 (49.1)	20 (57.1)	10 (45.5)	NS^a^
Age, mean (SD), yr	59.7(14.8)	59.6 (13.4)	65.6 (12.0)	50.0 (17.2)*	<0.001^b^
BMI, mean (SD), kg/m^2^	29.5 (5.0)	29.2 (4.89)	30.1 (5.2)	29.7 (5.2)	NS^c^
Macroadenoma, *n* (%)	91 (82.7)	43 (82.7)	28 (80.0)	20 (90.9)	NS^a^
Time since diagnosis, mean (SD), yr	10.8 (8.0)	10.2 (7.6)	12.4 (7.9)	9.7 (9.3)	NS^c^
Time from diagnosis to the beginning of					
treatment, mean (SD), months	23.39 (51.99)	19.56 (52.01)	36.01 (60.75)	14.90 (34.33)	NS^c^
IGF-1 levels at study visit					
within normal limits, *n* (%)	71 (64.0)	52 (98.1)	18 (51.4)**	0 (0.0)^**, ##^	<0.001^d^
>ULN, ≤ 1.2 x ULN, *n* (%)	18 (16.3)	1 (1.9)	17 (48.6)**	0 (0.0)^**, ##^	<0.001^d^
>1.2 x ULN, *n* (%)	22 (19.8)	0 (0.0)	0 (0.0)**	22 (100.0)^**, ##^	<0.001^d^
Tumour status					
Stable	102 (97.1)	49 (100.0)	31 (93.9)	21 (95.5)	NS^d^
Increase ≤20%	3 (2.9)	0 (0.0)	2 (6.1)	1 (4.5)	NS^d^
Increase >20%	0 (0.0)	0 (0.0)	0 (0.0)	0 (0.0)	NS^d^
Comorbidities					
Cardiac disease^1^, *n* (%)	60 (54.1)	26 (49.1)	21 (60.0)	12 (54.5)	NS^a^
Arthropathy, *n* (%)	47 (42.7)	22 (41.5)	18 (52.9)	7 (31.8)	NS^a^
Diabetes, *n* (%)	40 (36.0)	14 (26.4)	17 (48.6)	8 (36.4)	NS^a^
Apnea, *n* (%)	23 (20.9)	10 (18.9)	9 (25.7)	3 (14.3)	NS^a^
Hypopituitarism, *n* (%)	22 (19.8)	10 (18.9)	7 (20)	5 (22.7)	
phPASQ score (0–48), mean (SD)	12.9 (8.6)	10.4 (6.8)	16.4 (9.3)**	13.3 (9.6)	<0.05^c^
Headache score (0–8), mean (SD)	1.6 (1.9)	1.1 (1.5)	1.9 (2.0)	2.2 (2.2)*	<0.05^c^
Excessive sweating score (0–8), mean (SD)	1.9 (1.9)	1.8 (1.8)	2.1 (1.9)	2.0 (2.0)	NS^c^
Joint pain score (0–8), mean (SD)	3.0 (2.4)	2.4 (2.0)	4.1 (2.7)**	2.6 (2.3)^#^	<0.05^c^
Fatigue score (0–8), mean (SD)	2.7 (2.0)	2.2 (1.7)	3.6 (2.3)**	2.3 (1.7)^#^	<0.05^c^
Swelling score (0–8), mean (SD)	1.7 (1.6)	1.4 (1.4)	2.0 (1.6)	2.2 (2.0)	<0.05^b^
Numbness or tingling score (0–8), mean (SD)	2.0 (1.7)	1.6 (1.4)	2.6 (2.0)*	2.0 (1.7)	<0.05^b^
Health Status PASQ, mean (SD)	3.7 (2.2)	3.0 (1.5)	4.9 (2.6)**	3.6 (2.3)	<0.001
paPASQ score (0–48), mean (SD)	15.93 (10.99)	13.96 (9.93)	19.46 (11.75)	15.14 (11.43)	NS^c^
Headache score (0–8), mean (SD)	1.86 (2,14)	1.4 (1.88)	2.17 (2.28)	2.5 (2.39)	NS^c^
Excessive sweating score (0–8), mean (SD)	2.46 (2.49)	2.38 (2.43)	2.57 (2.58)	2.59 (2.61)	NS^c^
Joint pain score (0–8), mean (SD)	3.53 (2.75)	3.06 (2.55)	4.46 (2.95)	3.27 (2.68)	NS^c^
Fatigue score (0–8), mean (SD)	3.13 (2.58)	2.65 (2.42)	4.03 (2.79) *	2.86 (2.38)	<0.05^b^
Swelling score (0–8), mean (SD)	2.2 (2.4)	2.04 (2.15)	2.69 (2.7)	1.91 (2.45)	NS^c^
Numbness or tingling score (0–8), mean (SD)	2.75 (2.52)	2.52 (2.37)	3.54 (2.74)	2.0 (2.33)	NS^c^
Health Status PASQ, mean (SD)	4.22 (2.76)	3.49 (2.14)	5.51 (3.13) **	4.05 (2.84)	<0.01^c^
AcroQoL score (0–100), mean (SD)	65.7 (19.2)	70.5 (14.4)	56.9 (21.8)**	66.1 (22.1)	<0.01^b^
Physical (0–100) mean (SD)	59.9 (24.5)	67.6 (17.5)	46.3 (27.5)**	63.2 (25.8)^#^	<0.001^c^
Psychological (0–100), mean (SD)	68.0 (18.5)	71.8 (15.1)	61.4 (20.7)*	67.8 (21.1)	<0.05^b^
Appearance (0–100), mean (SD)	58.2 (22.3)	61.9 (19.9)	49.8 (23.4)*	60.6 (23.1)	<0.05^b^
Personal relations (0–100), mean (SD)	77.3 (18.5)	81.8 (13.6)	71.1 (21.1)*	75.0 (22.7)	<0.05^c^
Degree of disability, *n* (%)	26 (23.6)	11 (20.8)	11 (32.4)	4 (18.2)	NS^d^
<33%, *n* (%)	7 (28.0)	4 (36.4)	1 (10.0)	2 (50.0)	NS^d^
33–66%, *n* (%)	12 (48.0)	5 (45.5)	6 (60.0)	1 (25.0)	NS^d^
>66%, *n* (%)	6 (24.0)	2 (18.2)	3 (30.0)	1 (25.0)	NS^d^
Prior therapies					
Surgery, *n* (%)	84 (75.7)	44 (83.0)	25 (71.4)	15 (68.2)	NS^a^
Time since surgery, mean (SD), yr	11.2 (8.0)	10.5 (7.3)	14.0 (8.3)	8.9 (8.9)	NS^b^
Radiotherapy, *n* (%)	39 (35.1)	16 (30.2)	14 (40.0)	9 (40.9)	NS^a^
Time since radiotherapy, mean (SD), yr	10.3 (8.9)	10.8 (9.3)	11.5 (8.0)	7.1 (9.8)	NS^c^
Time in pharmacological treatment, mean (SD), yr	8.9 (6.9)	8.6 (5.9)	9.6 (7.1)	8.6 (9.0)	NS^c^
Number of medication changes in the last two years^2^					<0.001
None, *n* (%)	40 (38.1)	25 (50.0)	10 (28.6)	5 (25.0)^**, ##^	<0.001
1	28 (26.7)	12 (24.0)	16 (45.7)	0 (0.0)^**, ##^	<0.001
2–3, *n* (%)	31 (29.5)	10 (20.0)	7 (20.0)	14 (70.0)^**, ##^	<0.001
≥4, *n* (%)	6 (5.7)	3 (6.0)	2 (5.7)	1 (5.0)^**, ##^	<0.001
Time since last medication change, mean (SD), mo	28.1 (38.5)	33.7 (38.8)	32.5 (45.4)	8.5 (11.4)*	<0.05^c^
Time since last visit, mean (SD), mo	6.4 (3.8)	6.9 (3.9)	6.1 (3.6)	5.6 (4.0)	NS^c^

^1^includes hypertension, hyperlipidemia, or other cardiac abnormalities. ^2^Change of dose or treatment *p < 0.05 vs S. **p < 0.01 vs S. #*p* < 0.05 vs M-DA. ##*p* < 0.01 vs M-DA. NS: not significant. S: stable; M-DA: mild disease activity; S-DA: significant disease activity. a: Chi-square *p* value b: Anova p value; c: Kruskal-Wallis *p* value; d: Fisher *p* value

The most frequent co-morbidity was cardiac disease (54.1%), followed by arthropathy (42.7%), diabetes (36.0%) and sleep apnoea (20.9%) (Table 2). Main comorbidities were controlled in most patients. However, 11.5% of patients presented an uncontrolled cardiac disease and 14.3% an uncontrolled sleep apnoea.

Disability was present in 23.6% of patients (Table 2) and 8.8% of patients were on work leave at the study visit. Most patients had undergone pituitary surgery (75.7%), a third of patients (35.1%) received radiotherapy (34.2% of patients had undergone surgery and radiotherapy), and 23.4% of patients had not undergone surgery nor radiotherapy.

### Disease activity

**Fig. 1:**
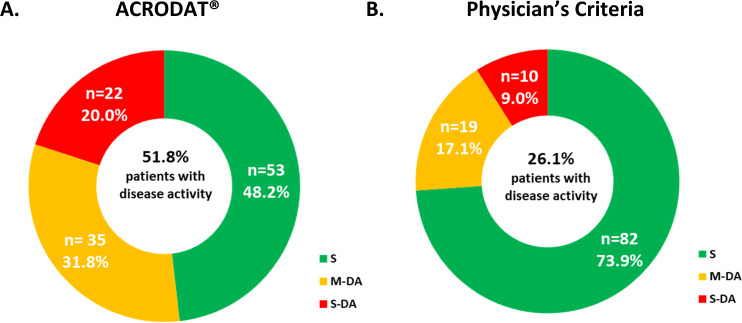
Disease activity according to ACRODAT^®^ (**A**) and Physicians’ Criteria (**B**). S stable, M-DA mild disease activity, S-DA significant disease activity

According to ACRODAT^®^, 48.2% of patients were classified as controlled (S) and 51.8% as having active disease: 31.8% M-DA and 20.0% S-DA (Fig. 1A).

**Fig. 2:**
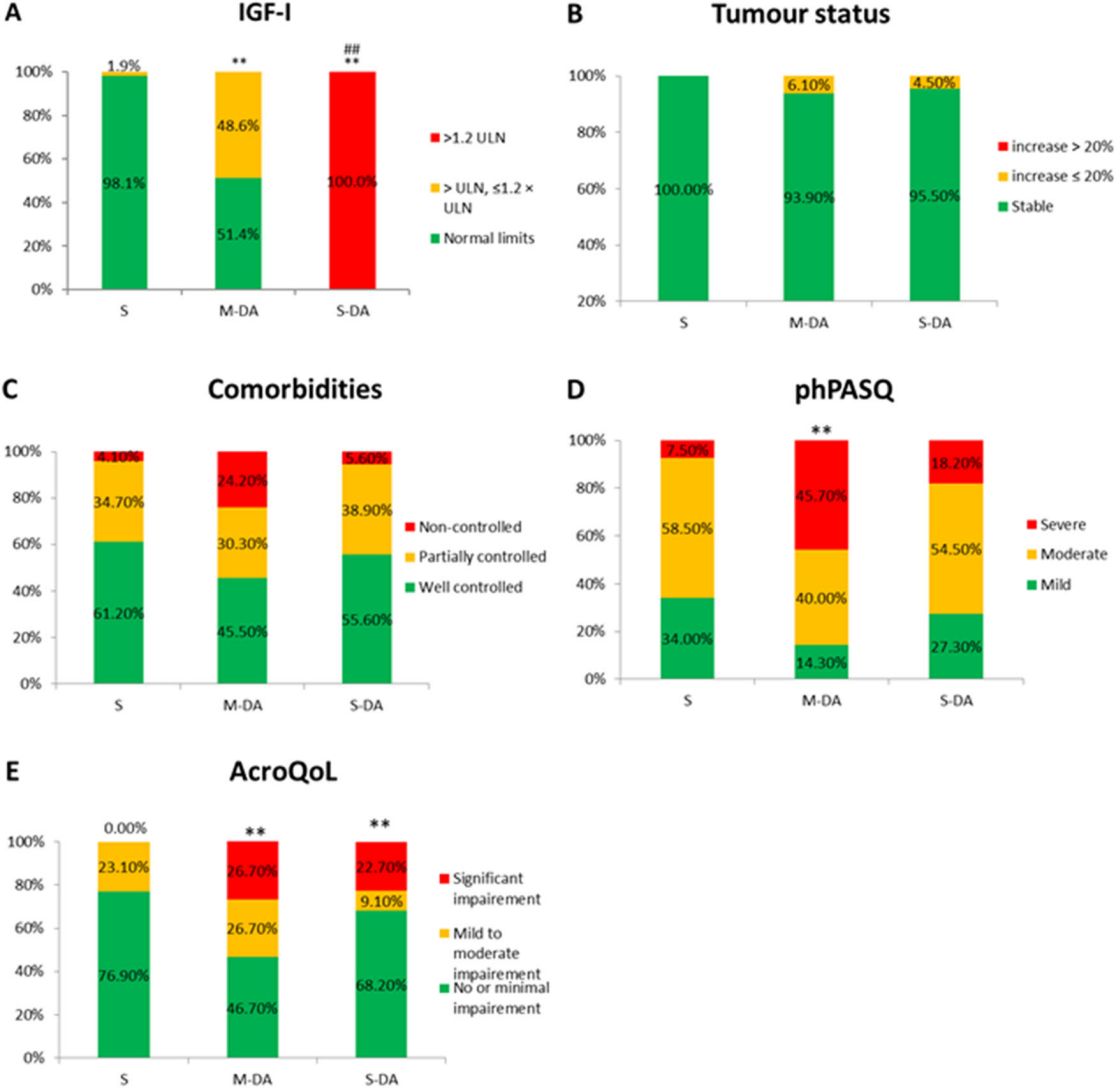
Patients’ characteristics according to ACRODAT^®^. **A** IGF-I: insulin-like growth factor-I. **B** Tumour status. **C** Comorbidities. **D** phPASQ: Physician-assessed Acromegaly Symptom Questionnaire fulfilled by the physician. **E** AcroQoL Acromegaly Quality of Life Questionnaire. S stable, M-DA mild disease activity, S-DA significant disease activity; *p < 0.01 vs. S; **p < 0.001 vs. S; ##*p* < 0.001 vs. M-DA

S-DA patients were significantly younger, had a significantly higher headache severity, had a significantly higher number of medication changes in the last two years and their last change in medication was significantly more recent compared to S patients (Table 2). Both M-DA and S-DA patients presented significantly higher values of IGF-I compared with S patients (*p* < 0.001) (Table 2, Fig. 2A). Consistently with the algorithm behind ACRODAT^®^, 100% of S-DA patients had IGF-I levels >1.2 ULN (Table 2, Fig. 2A). There were no statistically significant differences in comorbidities or tumour status among S, M-DA or S-DA patients (Table 2, Fig. 2B, C). Symptoms (phPASQ) were significantly more severe (*p* = 0.004) and QoL significantly worse (*p* = 0.005) in M-DA patients compared to S patients, and, in some items (joint pain and fatigue severity and physical component of AcroQoL) compared to S-DA patients (Table 2, Fig. 2D, E).

According to physicians’ clinical evaluation, 73.9%, 17.1% and 9.0% were classified as S, M-DA or S-DA respectively (Fig. 1B). ACRODAT^®^ classification of disease activity significantly correlated with physicians’ opinion (Pearson’s correlation coefficient 0.621, *p* < 0.001), with a moderate inter-rater agreement [kappa agreement coefficient 0.569; 95% confidence interval (CI_95%_): 0.402–0.678]. Predictive analysis between controlled (S) or active disease (M-DA + S-DA) groups according to ACRODAT vs. physicians’ opinion showed a fair inter-rater agreement (kappa agreement 0.356; CI_95%_ 0.207–0.506) a specificity value of 92.5% (CI_95%_: 84.4–100), sensitivity 43.9% (CI_95%_: 30.1–57.6), PPV 86.2% (CI_95%_: 71.9–100) and NPV 60.5% (CI_95%_: 49.2–71.8).

Discrepancies between ACRODAT^®^ classification of disease activity and physicians’ criteria were observed in patients with IGF-I levels above ULN (*p* < 0.001), patients with higher symptomatology [joint pain (*p* < 0.05), numbness or tingling (*p* < 0.05) and global symptomatology (*p* < 0.01) according to phPASQ, fatigue (*p* < 0.05) according to paPASQ], worse health status [according to paPASQ (*p* < 0.001) and phPASQ (*p* < 0.01)] and patients showing more QoL impairment (*p* < 0.05). Moreover, discrepancies were also observed in patients with longer time since diagnosis (*p* < 0.05) or since the beginning of treatment (*p* < 0.05). A univariate analysis confirmed these results (Supplementary Table 1). A multivariant analysis showed that the existence of discrepancies between ACRODAT^®^ classification and physicians’ criteria relied on IGF-I, phPASQ and time since diagnosis (*p* < 0.05) (Supplementary Table 2).

### Quality of life

Overall, patients reported mild impairment in their QoL with a mean (SD) AcroQoL total score of 65.7 (19.2) (Tables 2 and 3). Patients scored worse in the physical domain and in the psychological-appearance domain (Tables 2 and 3).

**Table 3:** Normalized (0–100) AcroQoL overall score and subscores included in the AcroQoL questionnaire

	Normalized score (0–100)	
	Mean	SD
Physical score	59.94	24.46
My legs feel weak	63.7	29.1
I get depressed	63.6	28.9
I have problems carrying out my usual activities (e.g., working, studying, doing household task, family or leisure activities)	64.3	31.9
The illness affects my performance at work or in my usual tasks	63.5	34.2
My joints ache	49.3	32.2
I feel tired	50	27.8
I feel like a sick person	65.1	33.8
I feel weak	59.7	30.2
Psychological score	68.01	18.5
Appearance score	58.215	22.30
I feel ugly	57.4	33.3
I look awful in photographs	46.6	34.6
I look different in the mirror	57.4	33.6
Some parts of my body (nose, feet, hands, etc.) are too big	49.1	36.5
I have problems doing things with my hands, for example, sewing or handling tools	68.0	32.1
I snore at night	47.7	31.7
It is hard for me to articulate words due to the size of my tongue	81.3	25.1
Personal relations score	77.31	18.52
I avoid going out very much with friends because of my appearance	89.4	20.1
I try to avoid socializing	84.5	23.1
I feel rejected by people because of my illness	90.5	20.0
People stare at me because of my appearance	82.0	26.8
I have problems with sexual relations	69.6	33.6
The physical changes produced by my illness govern my life	68.2	33.2
I have little sexual appetite	53	34.3
Total score	65.67	19.23

According to ACRODAT^®^ levels of severity for QoL (Table 1), 33.7% of patients presented a mild to moderate (21.2%) or significant (12.5%) impairment on QoL.

No correlation was found between IGF-I levels and AcroQoL (Pearson’s correlation coefficient 0.084, *p* = 0.395).

Among the 65 patients (60.7%) with controlled IGF-I and tumour status (IGF-I < ULN and tumour not visible or without changes), 1 (1.5%) and 7 (10.8%) presented a significant and mild to moderate impairment of QoL respectively. These patients presented a significant higher time since diagnosis than patients with no or minimal impairment of QoL (mean ± SD = 25.85 ± 0 years in patients with significant impairment; 14.57 ± 5.16 years in patients with mild to moderate impairment; 10.34 ± 7.76 in patients with no or minimal impairment; p = 0.048).

### Symptoms

Overall, patients suffered from mild-moderate acromegaly symptoms [mean (SD) phPASQ total score 12.9 (8.6)] (Table 4). According to ACRODAT^®^ levels of severity for symptoms (Table 1), 73,6% of patients showed moderate (51.8%) or severe (21.8%) symptoms.

**Table 4:** Agreement on symptoms severity between patients (paPASQ) and physicians (phPASQ)

	phPASQ	paPASQ	Pearson coefficient	*p*	kappa
Headache, mean (SD)	1.5 (1.9)	1.9 (2.1)	0.754	<0.001	0.726
Excessive sweating, mean (SD)	1.9 (1.9)	2.5 (2.5)	0.773	<0.001	0.625
Joint pain, mean (SD)	3.0 (2.4)	3.5 (2.7)	0.752	<0.001	0.641
Fatigue, mean (SD)	2.7 (2.0)	3.1 (2.6)	0.641	<0.001	0.570
Swelling, mean (SD)	1.7 (1.6)	2.2 (2.4)	0.584	<0.001	0.327
Numbness or tingling, mean (SD)	2.0 (1.7)	2.7 (2.5)	0.691	<0.001	0.505
Total PASQ (symptoms) score, mean (SD)	12.9 (8.6)	15.9 (11.0)	0.759	<0.001	0.624
Health Status PASQ, mean (SD)	3.7 (2.2)	4.2 (2.8)	0.749	<0.001	0.662

Each item was scored on a 9-point scale (0 no symptoms-8 severe incapacitating symptoms). The total PASQ score was the sum of the individual symptom scores (maximum = 48). Overall health status was scored ranging from 0 (best possible) to 10 (worst possible)

*PASQ* patient-assessed acromegaly symptom questionnaire

Physicians rated patient symptoms significantly lower in severity than patients (*p* < 0.001). However, phPASQ significantly correlated with paPASQ with a substantial inter-rater agreement in both PASQ total score and individual symptoms sub-scores (Table 4). No characteristic traits in patients with discrepancies between phPASQ and paPASQ were found.

No correlation was found between IGF-I levels and phPASQ (Pearson’s correlation coefficient: 0.049, *p* = 0.615) or paPASQ (Pearson’s correlation coefficient: −0.011, *p* = 0.911) neither in the total score nor in symptoms or health status sub-scores.

Among the 65 patients (60.7%) with controlled IGF-I and tumour status (IGF-I < ULN and tumour not visible or without changes), 13 (20.0%) and 34 (52.3%) presented severe and moderate symptoms respectively according to their phPASQ (32.3 % and 53.8% respectively according to their paPASQ). No characteristic demographic traits were found for these subgroups of patients.

### Therapeutic action

Overall, a therapeutic action regarding acromegaly management was taken in 28.0% of patients in the in the visit previous to the study visit. A significantly higher rate of action was taken on patients with IGF-I > 1.2 ULN than in patients with normal IGF-I (71.4% vs 13.4%; *p* < 0.001) or IGF-I > ULN but <1.2 ULN at the visit before study visit (71.4% vs 33.3%; *p* < 0,01).

Results of the therapeutic action taken in the previous visit was not successful in most of the patients: 88.9% (*n* = 16) of patients with no IGF-I and/or tumour control, remained uncontrolled at the study visit. Similarly, 82.8% (*n* = 53) of patients in which no action was taken in the last visit remained in the same status. Most of them (83.9%, *n* = 47) were controlled and remained controlled. Fourteen point one % of patients (*n* = 9) who were controlled in terms of IGF-I control and tumour at prior visit, spontaneously lost control at the study visit.

**Fig. 3:**
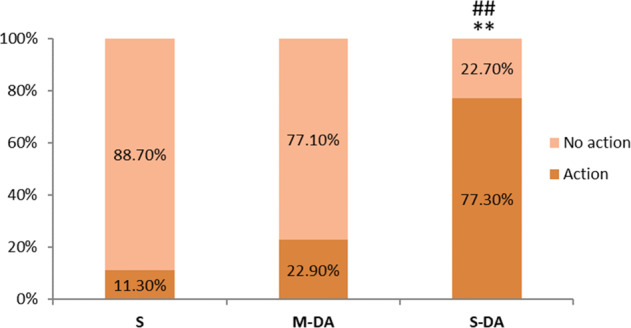
Therapeutic action taken depending on ACRODAT^®^ classification at study visit. S stable, M-DA mild disease activity, S-DA significant disease activity. **p < 0.001 vs. S; ##*p* < 0.001 vs. M-DA

At the study visit, therapeutic action was taken in 27.9% of patients overall and a change of medication (dose or drug) was the most frequent action taken (20,7% of patients) (Table 5). In S-DA patients, therapeutic actions were taken on a significantly greater proportion (77.3%) compared to M-DA (22.9%, *p* < 0.001) and S patients (11.3%, *p* < 0.001) (Fig. 3). However, no action was taken on 22.7% (*n* = 5) of S-DA patients and in 77.1% (*n* = 27) of M-DA patients.

**Table 5:** Therapeutic action taken at the study visit

	Total	S	M-DA	S-DA	*p* value
Action	31 (27.9)	6 (11.3)	8 (22.9)	17 (77.3)^**,#^	<0.001
Surgery evaluation	6 (5.4)	2 (3.8)	2 (5.7)	2 (9.1)	
Change of medication (dose or drug)	23 (20.7)	4 (7.5)	6 (17.1)	13 (59.1)	
Radiotherapy	2 (1.8)	0 (0.0)	0 (0.0)	2 (9.1)	
No action	80 (72.1)	47 (88.7)	27 (77.1)	5 (22.7)^**, #^	

*S* stable, *M-DA* mild disease activity, *S-DA* significant disease activity

**p < 0.001 vs S. #*p* < 0.01 vs M-DA

In 3 of those 5 S-DA patients, a change in treatment (dose or drug) was taken in the last visit, that took place a mean (SD) of 5.2 (2.7) months before the study visit. In the other 2 patients, no action was taken either in the last visit or in the study visit. Both presented IGF-I levels above ULN at both visits, but one of them was considered stable according to physician criteria.

Differences between M-DA patients in which no action was taken (*n* = 27) vs patients in which action was taken (*n* = 8) at the study visit were found in patients where the disease activity classification by physician’s criteria was different than ACRODAT® classification (4 vs 24 classified as S-DA, *p* < 0.05), patients with longer time since the beginning of treatment (mean time 5.87 vs 10.41 years, *p* < 0.05) and patients with longer time since the last visit (mean time 3.96 vs 6.70 years, *p* < 0.05).

Criteria that marked the decision to take a therapeutic action on S-DA and M-DA patients were IGF-I levels and IGF-I + tumour control at the last visit and at the study visit (*p* < 0.01).

## Discussion

Traditional clinical treatment goals for acromegaly are mainly based on the achievement of biochemical and tumour control [6, 10]. However, even when biochemical and tumour control is achieved, many patients continue to experience symptoms and impaired HRQoL [6, 12, 13, 21]. Moreover, challenges in the IGF-I measurement [22, 23] and subjectivity in tumour growth assessment [24] limits the use of these parameters as the sole assessment of disease activity.

ACRODAT^®^ is a new software medical device designed to assess acromegaly activity from an integrated point of view as it includes not only clinical parameters of disease but also patients’s reported outcomes (PRO), such as symptoms and HRQoL [18]. AcroVoice, a study to determine which parameters matter to acromegaly patients for defining their disease, showed that, in contrast with the physician validation study, patients placed more value on the “patient-centred” parameters [6]. Thus, validated PRO should be regularly documented in acromegaly patients as a patient-oriented indicator of treatment success [13, 15]. Indeed, objective tools such as ACRODAT^®^ and SAGIT® have been recently recommended to assess and monitor acromegaly disease activity [1, 25, 26].

Herein, we show the first real world data evaluating disease activity status using ACRODAT^®^ and its correlation with physicians’ criteria, in a representative cohort of 111 Spanish patients attended in a clinical practice setting. Patient population was similar to other studies [27, 28], with a mean age of 59.7 years and 51.4% of women.

60.7% of patients were controlled in terms of IGF-I and tumour volume. According to ACRODAT^®^, only 48.2% of patients were considered controlled or stable and 51.8% presented with active disease (Fig. 1A). According to the physicians’ clinical evaluation, 73.9% of patients were stable whereas 26.1% presented with uncontrolleddisease (Fig. 2B). ACRODAT^®^ and physician’s opinion showed a fair inter-rater agreement. Thus, for patients who according to the physician were stable, 92.5% of them were classified by ACRODAT^®^ as stable. However, ACRODAT^®^ was able to identify more cases of active disease due to the integration of other than biochemical and tumour control parameters such as comorbidities, symptoms and QoL in its definition of control. Discrepancies between ACRODAT^®^ and physicians’ criteria were mainly observed in patients with IGF-I above ULN, phPASQ (more severe signs and symptoms), patients showing more QoL impairment and a longer time since diagnosis or since the beginning of treatment. Thus, ACRODAT® may be especially useful in determining disease activity in patients with these characteristics. A source of discrepancy may be the existing differences in the definition of biochemical control: while ACRODAT^®^ considers an IGF-I level >1 ULN as active disease, the currently used cut-offs in clinical practice ranges from 1 to 1.5 ULN [29]. A possible reason is the distrust in the accuracy of IGF-I levels due to the variability of the diagnostic assays [22, 23, 30]. However, all-cause mortality risk in acromegaly increases with higher serum IGF-I levels and that IGF-I normalization (IGF-I < ULN) is associated with all-cause mortality rates indistinguishable from the general population [31].

IGF-I levels do not correlate with symptoms or QoL as has been described in this and other studies [14, 15]. In fact, we found that 12.3% of patients with IGF-I and tumour control had a mild to moderate or significant impairment in QoL. Similarly, 72.3% of patients according to phPASQ, or 86.1% according to paPASQ, showed moderate or severe symptoms despite having reached IGF-I and tumour control. Interestingly, patients with IGF-1 and tumour control that had impairment in QoL and symptoms according to paPASQ did not statistically differences in the time since diagnosis in comparison with patients with IGF-1 and tumour control that showed stable QoL and mild symptomatology (data not shown). phPASQ and paPASQ showed a substantial inter-rater agreement, however, physicians tended to rate the intensity of symptoms significantly lower than patients. These findings support the need to consider validated PROs in disease activity assessment although, currently, this is not common clinical practice: 53.8% and 30.8% of the physicians involved in this study affirmed not using PASQ and AcroQoL, respectively (data not shown). The study also showed that there were a proportion of patients with uncontrolled cardiac disease (11.5%) or sleep apnoea (14.3%). Comorbidities should be regularly monitored and appropriate therapeutic actions should be taken to minimize disease burden and mortality.

Most patients can achieve disease control if their treatment is adequately selected and adjusted according to patient characteristics and response [32]. Indeed, Ragonese et al. showed that, when adequately titrated, pegvisomant could achieve IGF-I control in 89.6% patients [33], a higher proportion than reported whentitration was applied in usual clinical practise as described in Acrostudy [24]. In our study, therapeutic actions were taken in a significantly greater proportion of S-DA patients compared to M-DA and S patients, but no action was taken in a significant number of patients with disease activity according to ACRODAT^®^ (22.7% of S-DA patients and 77.1% of M-DA patients) (Fig. 3). A possible explanation may be that these patients were in the process of treatment adjustment (S-DA patients had more changes of medication and more recently) and physicians were waiting to see a stabilization before making a decision. Time between visits in these cases should be optimized. Discrepancies in the IGF-I cut-offs levels or the low reliability of IGF-I determinations may explain the inaction on some M-DA patients. Indeed, Schöfl et al. [30], described that one of the main reasons not to change/escalate treatment in uncontrolled patients was the fluctuating IGF-I levels. In any case, there were a significant number of patients with IGF-I and tumour control but poor PROs that did not receive a possible change in treatment that they might have needed. Furthermore, circulating IGF-I does not guarantee normal exposure to IGF-I in all tissues as demonstrated by Neggers et al. [12]. The lack of therapeutic action may also be a consequence of clinical inertia such as “watch and wait” or “trial-error” attitude. Therapeutic decision in S-DA and M-DA patients was guided by IGF-I levels and IGF-I+ tumour control despite collecting patient PROs in the study visit, showing that that clinical and analytical data collection may not be useful if it is not integrated and interpreted. ACRODAT^®^ may support the decision-making process by providing an integrated holistic view of the disease, even though in some cases it might be difficult to obtain all PROs in the time frame of a routine outpatient visit.

Some limitations derived from the cross-sectional nature of this study should be borne in mind. The data presented here are a cross-sectional picture of a specific moment of the state of illness of patients with a long evolution of the disease. ACRODAT^®^ is designed to long-term monitor changes at regular intervals that can facilitate a better management of patients. Second, the PASQ used in ACRODAT^®^ in this study was completed by physicians, although concordance was observed between the PASQ completed both by patients and physicians. Finally, therapeutic action collected in the study referred to acromegaly treatments, and specific treatment for comorbidities or symptoms were not collected.

In conclusion, ACRODAT^®^ is a validated and highly specific tool that allows routinely monitoring of disease activity in a holistic manner by incorporating clinical, laboratory and radiological parameters (IGF-I, tumour status and comorbidities) as well as PRO parameters such as PASQ and AcroQoL. Monitoring changes at regular intervals may be useful to identify patients with non-obvious disease activity, facilitate better treatment decisions and support an integral approach to acromegaly disease management.

## Supplementary Information

Supplementary Information

Supplementary Information
