# Supplementary material for: Acromegaly disease activity according to ACRODAT®, a cross-sectional study in Spain: ACROVAL study
Source: Endocrine. 2021 Oct 19;75(2):525–36. doi: 10.1007/s12020-021-02900-0 (PMC8816757; doi:10.1007/s12020-021-02900-0)
Supplement: Supplementary file 1 — Supplementary Information [file 12020_2021_2900_MOESM1_ESM.docx]

**Supplementary Table 1. Associated factors to the discrepancy between ACRODAT® and physisician’s criteria. A univariate analysis.** The OR (Odds Ratio) determines the association of each of the risk factors with the dependent variable in an independent manner eliminating the possibility that another factor confuses the effect of another. The constant is a part of the model that is self-explanatory regardless of the factors included.

| **Parameters** | | **OR_Crude_** | **95% C.I. for OR_Crude_** | | **p-value** |
| --- | --- | --- | --- | --- | --- |
|  |  |  | **Inf** | **Sup** |  |
| **IGF-I levels in the**  **current visit** | Levels are within normal limits |  |  |  | 0 |
|  | The levels exceed a maximum of 1.2 times the ULN or when the levels are below the limit | 9.579 | 2.802 | 32.744 | 0.000 |
|  | IGF-1 levels are significantly elevated and exceed 1.2 times the ULN | 3.953 | 1.455 | 10.737 | 0.007 |
|  | Constant | 0.365 |  |  | 0 |
| **Current visit: Variation in tumor size since the last visit (Tumor is not visible or has not changed in volume)** | Slight increase in size (<= 20%) | 2.976 | 0.261 | 33.897 | 0.38 |
|  | Constant | 0.672 |  |  | 0.049 |
| **Last visit: Variation in tumor size since the last visit (Tumor is not visible or has not changed in volume)** | Slight increase in size (<= 20%) | 2349781590 | 0 | - | 0.999 |
|  | Constant | 0.687 |  |  | 0.056 |
| **Comorbidities** | Missing |  |  |  | 0.502 |
|  | Moderate | 1.556 | 0.652 | 3.71 | 0.319 |
|  | Significant | 1.75 | 0.497 | 6.157 | 0.383 |
|  | Constant | 0.571 |  |  | 0.046 |
| **phPASQ** | Mild | |  |  | 0.008 |
|  | Moderate | 0.95 | 0.37 | 2.44 | 0.915 |
|  | Severe | 4.614 | 1.437 | 14.817 | 0.01 |
|  | Constant | 0.526 |  |  | 0.1 |
| **Symptomatology, Headache** | Low | |  |  | 0.112 |
|  | Regular | 2.473 | 0.979 | 6.249 | 0.055 |
|  | High | 3.533 | 0.307 | 40.614 | 0.311 |
|  | Constant | 0.566 |  |  | 0.013 |
| **Symptomatology, Excessive sweating (according to phPASQ)** | Low | |  |  | 0.808 |
|  | Regular | 0.791 | 0.349 | 1.792 | 0.575 |
|  | High | 1.3 | 0.173 | 9.77 | 0.799 |
|  | Constant | 0.769 |  |  | 0.28 |
| **Symptomatology, Joint pain (according to phPASQ)** | Low | |  |  | 0.064 |
|  | Regular | 1.287 | 0.556 | 2.976 | 0.555 |
|  | High symptomatology | 4.625 | 1.285 | 16.647 | 0.019 |
|  | Constant | 0.541 |  |  | 0.027 |
| **Symptomatology, Fatigue (according to phPASQ)** | Low | |  |  | 0.133 |
|  | Regular | 1.917 | 0.866 | 4.24 | 0.108 |
|  | High | 4 | 0.672 | 23.826 | 0.128 |
|  | Constant | 0.5 |  |  | 0.014 |
| **Symptomatology, Swelling (according to phPASQ)** | Low | |  |  |  |
|  | Regular | 0.867 | 0.377 | 1.99 | 0.736 |
|  | High | - | - |  | - |
|  | Constant | 0.75 |  |  | 0.212 |
| **Symptomatology, Numbness or tingling of the extremities (according to phPASQ)** | Low | |  |  | 0.031 |
|  | Regular | 3.062 | 1.33 | 7.052 | 0.009 |
|  | High | 0 | 0 | . | 1 |
|  | Constant | 0.49 |  |  | 0.004 |
| **Assessment of health status according to the symptoms mentioned (according to phPASQ)** | Good^(*)^ |  |  |  | 0.002 |
|  | Regular | 2.344 | 0.961 | 5.717 | 0.061 |
|  | Bad | 10.833 | 2.755 | 42.6 | 0.001 |
|  | Constant | 0.4 |  |  | 0.001 |
| **paPASQ** | Mild | |  |  | 0.24 |
|  | Moderate | 0.556 | 0.192 | 1.609 | 0.279 |
|  | Severe | 1.111 | 0.369 | 3.346 | 0.851 |
|  | Constant | 0.9 |  |  | 0.819 |
| **Symptomatology, Headache (according to paPASQ)** | Low | |  |  | 0.083 |
|  | Regular | 2.47 | 1.046 | 5.835 | 0.039 |
|  | High | 3.778 | 0.328 | 43.576 | 0.287 |
|  | Constant | 0.529 |  |  | 0.008 |
| **Symptomatology, Excessive sweating (according to paPASQ)** | Low | |  |  | 0.805 |
|  | Regular | 1.263 | 0.551 | 2.896 | 0.581 |
|  | High | 0.857 | 0.228 | 3.224 | 0.82 |
|  | Constant | 0.667 |  |  | 0.109 |
| **Symptomatology, Joint pain (according to paPASQ)** | Low | |  |  | 0.635 |
|  | Regular | 1.2 | 0.517 | 2.784 | 0.671 |
|  | High | 1.667 | 0.581 | 4.779 | 0.342 |
|  | Constant | 0.6 |  |  | 0.087 |
| **Symptomatology, Fatigue (according to paPASQ)** | Low | |  |  | 0.025 |
|  | Regular | 0.914 | 0.398 | 2.099 | 0.832 |
|  | High | 6.111 | 1.501 | 24.881 | 0.012 |
|  | Constant | 0.6 |  |  | 0.087 |
| **Symptomatology, Swelling (according to paPASQ)** | Low | |  |  | 0.186 |
|  | Regular | 0.533 | 0.221 | 1.286 | 0.162 |
|  | High | 2.043 | 0.452 | 9.229 | 0.353 |
|  | Constant | 0.816 |  |  | 0.4 |
| **Symptomatology, Numbness or tingling of the extremities (according to paPASQ)** | Low | |  |  | 0.309 |
|  | Regular | 1.682 | 0.745 | 3.796 | 0.211 |
|  | High | 2.22 | 0.602 | 8.191 | 0.231 |
|  | Constant | 0.541 |  |  | 0.027 |
| **Assessment of health status according to the symptoms mentioned (according to paPASQ)** | Good |  |  |  | 0.007 |
|  | Regular | 1.072 | 0.439 | 2.616 | 0.879 |
|  | Bad | 5.019 | 1.714 | 14.7 | 0.003 |
|  | Constant | 0.484 |  |  | 0.021 |
| **Time since acromegaly diagnosis (years)** | Time (Years) | 1.064 | 1.012 | 1.12 | 0.016 |
|  | Constant | 0.35 |  |  | 0.002 |
| **Time from diagnosis to start the treatment (years)** | Time from diagnosis to start the treatment (years) | 1.066 | 1.006 | 1.131 | 0.031 |
|  | Constant | 0.384 |  |  | 0.005 |
| **Time since last visit** | Time since last visit | 0.947 | 0.853 | 1.052 | 0.311 |
|  | Constant | 0.994 |  |  | 0.988 |
| **Patient age at study visit (years)** | Patient age at study visit (years) | 1.001 | 0.976 | 1.027 | 0.937 |
|  | Constant | 0.665 |  |  | 0.613 |
| **Gender: Male** | Gender (Female) | 1.058 | 0.497 | 2.252 | 0.884 |
|  | Constant | 0.688 |  |  | 0.176 |

IGF-I: insulin-like growth factor-I; PASQ: Patient acromegalic symptom questionnaire; phPASQ; Patient acromegalic symptom questionnaire (fulfilled by physicians); paPASQ; Patient acromegalic symptom questionnaire (fulfilled by patients); ULN: Upper Limit of Normality.
