# Supplementary material for: Acromegaly disease activity according to ACRODAT®, a cross-sectional study in Spain: ACROVAL study
Source: Endocrine. 2021 Oct 19;75(2):525–36. doi: 10.1007/s12020-021-02900-0 (PMC8816757; doi:10.1007/s12020-021-02900-0)
Supplement: Supplementary file 2 — Supplementary Information [file 12020_2021_2900_MOESM2_ESM.docx]

**Supplementary Table 2. Associated factors for the discrepancy between ACRODAT ® and the physisican’s criteria. A multivariate model.** The OR (Odds Ratio) determines the association of each of the risk factors with the dependent variable in an independent manner eliminating the possibility that another factor confuses the effect of another. The constant is a part of the model that is self-explanatory regardless of the factors included.

| **Parameters** | | **OR_Ajusted_** | **95% C.I. for OR_Adjusted_** | | **p-value** |
| --- | --- | --- | --- | --- | --- |
|  |  |  | **Inf.** | **Sup.** |  |
| **IGF-I level in the current visit** | Levels are within normal limits |  |  |  | 0 |
|  | The levels exceed a maximum of 1.2 times the ULN or when the levels are below the limit | 16.531 | 3.368 | 81.13 | 0.001 |
|  | IGF-I levels are significantly elevated and exceed 1.2 times the ULN | 6.903 | 2.082 | 22.88 | 0.002 |
| **phPASQ: Assessment of health status according to the symptoms mentioned** | Good |  |  |  | 0.001 |
|  | Regular | 3.269 | 1.074 | 9.947 | 0.037 |
|  | Bad | 25.194 | 4.328 | 146.7 | 0 |
| **Time from diagnosis to start the treatment (years)** | | 1.089 | 1.023 | 1.16 | 0.008 |
| **Constant** | | 0.052 |  |  | 0 |

IGF-I: insulin-like growth factor-I; PASQ: Patient acromegalic symptom questionnaire: phPASQ; Medical patient acromegalic symptom questionnaire (fulfilled by the physician); ULN: Upper Limit of Normality
